# Statins and cognitive decline in patients with Alzheimer’s and mixed dementia: a longitudinal registry-based cohort study

**DOI:** 10.1186/s13195-023-01360-0

**Published:** 2023-12-20

**Authors:** Bojana Petek, Henrike Häbel, Hong Xu, Marta Villa-Lopez, Irena Kalar, Minh Tuan Hoang, Silvia Maioli, Joana B. Pereira, Shayan Mostafaei, Bengt Winblad, Milica Gregoric Kramberger, Maria Eriksdotter, Sara Garcia-Ptacek

**Affiliations:** 1 Division of Neurogeriatrics, Department of Neurobiology, Care Sciences and Society, Karolinska Institutet, Stockholm, Sweden; 2 Clinical Institute of Genomic Medicine, University Medical Centre Ljubljana, Ljubljana, Slovenia; 3 Faculty of Medicine, University of Ljubljana, Ljubljana, Slovenia; 4 Medical Statistics Unit, Department of Learning, Informatics, Management and Ethics, Karolinska Institutet, Stockholm, Sweden; 5 Division of Clinical Geriatrics, Department of Neurobiology, Care Sciences and Society, Karolinska Institutet, Stockholm, Sweden; 6 Faculty of Medicine, University Complutense of Madrid, Madrid, Spain; 7 Department of Neurology, University of Alberta Hospital, Edmonton, Canada; 8 Department of Neurology, University Medical Centre Ljubljana, Ljubljana, Slovenia; 9 Department of Medical Epidemiology and Biostatistics, Karolinska Institutet, Stockholm, Sweden; 10 Center for Alzheimer Research, Department of Neurobiology, Care Sciences and Society, Division of Neurogeriatrics, Karolinska Institutet, Stockholm, Sweden; 11 Division of Neuro, Department of Clinical Neurosciences, Karolinska Institutet, Stockholm, Sweden; 12 Aging and Inflammation Theme, Karolinska University Hospital, Stockholm, Sweden

**Keywords:** Cohort study, Statins, HMG-CoA reductase inhibitors, Alzheimer's disease, Cognitive impairment, Cholesterol metabolism

## Abstract

**Background:**

Disturbances in brain cholesterol homeostasis may be involved in the pathogenesis of Alzheimer’s disease (AD). Lipid-lowering medications could interfere with neurodegenerative processes in AD through cholesterol metabolism or other mechanisms.

**Objective:**

To explore the association between the use of lipid-lowering medications and cognitive decline over time in a cohort of patients with AD or mixed dementia with indication for lipid-lowering treatment.

**Methods:**

A longitudinal cohort study using the Swedish Registry for Cognitive/Dementia Disorders, linked with other Swedish national registries. Cognitive trajectories evaluated with mini-mental state examination (MMSE) were compared between statin users and non-users, individual statin users, groups of statins and non-statin lipid-lowering medications using mixed-effect regression models with inverse probability of drop out weighting. A dose-response analysis included statin users compared to non-users.

**Results:**

Our cohort consisted of 15,586 patients with mean age of 79.5 years at diagnosis and a majority of women (59.2 %). A dose-response effect was demonstrated: taking one defined daily dose of statins on average was associated with 0.63 more MMSE points after 3 years compared to no use of statins (95% CI: 0.33;0.94). Simvastatin users showed 1.01 more MMSE points (95% CI: 0.06;1.97) after 3 years compared to atorvastatin users. Younger (< 79.5 years at index date) simvastatin users had 0.80 more MMSE points compared to younger atorvastatin users (95% CI: 0.05;1.55) after 3 years. Simvastatin users had 1.03 more MMSE points (95% CI: 0.26;1.80) compared to rosuvastatin users after 3 years. No differences regarding statin lipophilicity were observed. The results of sensitivity analysis restricted to incident users were not consistent.

**Conclusions:**

Some patients with AD or mixed dementia with indication for lipid-lowering medication may benefit cognitively from statin treatment; however, further research is needed to clarify the findings of sensitivity analyses.

**Supplementary Information:**

The online version contains supplementary material available at 10.1186/s13195-023-01360-0.

## Background

The brain houses about a quarter of the cholesterol present in the body, making it the richest cholesterol-containing organ [1]. The essential role of brain cholesterol is reflected in its involvement in numerous physiological processes such as maintaining membrane integrity, neurotransmission and synaptogenesis [2]. A dysregulation of brain cholesterol homeostasis may be involved in the pathogenesis of Alzheimer’s disease [2] through interference with the amyloidogenic Aβ pathway [3], impairment of cerebral blood flow [4], and other mechanisms [5]. On the other hand, the association of peripheral hypercholesterolemia and cognition is complex. Peripheral hypercholesterolemia in midlife has been linked to cognitive decline and AD in late-life [6, 7] through different mechanisms [7–10]. Moreover, genetic polymorphism of brain cholesterol transporter ApoE4 and several additional genetic factors implicated in lipid metabolism could be relevant to AD pathogenesis [11, 12]. In contrast, peripheral hyperlipidaemia in late life is a marker of a better general health and cognition [13, 14].

The possible cognitive effects of HMG-CoA reductase inhibitors or statins, which are used in cardiovascular disease prevention, have sparked extensive research in the last few decades. Based on their pharmacokinetic characteristics, statins can be divided according to their structure (fungus-derived or synthetical), lipophilicity, metabolism, bioavailability, potency and binding to different proteins and transporters [15]. The multi-layered effects of statins on cognition are translated through numerous neurodegenerative processes in a cholesterol-dependent as well as independent (´´pleiotropic´´) manner [15, 16]. Statins seem to interfere with the amyloidogenic cascade [17] and phosphorylation of tau [18], provide beneficial vascular factors through endothelial function and clearance of neurotoxic substances [19], decrease neuroinflammation and oxidative stress as well as promote neuronal survival and plasticity, synaptogenesis and neurotransmission [16].

**Fig. 1:**
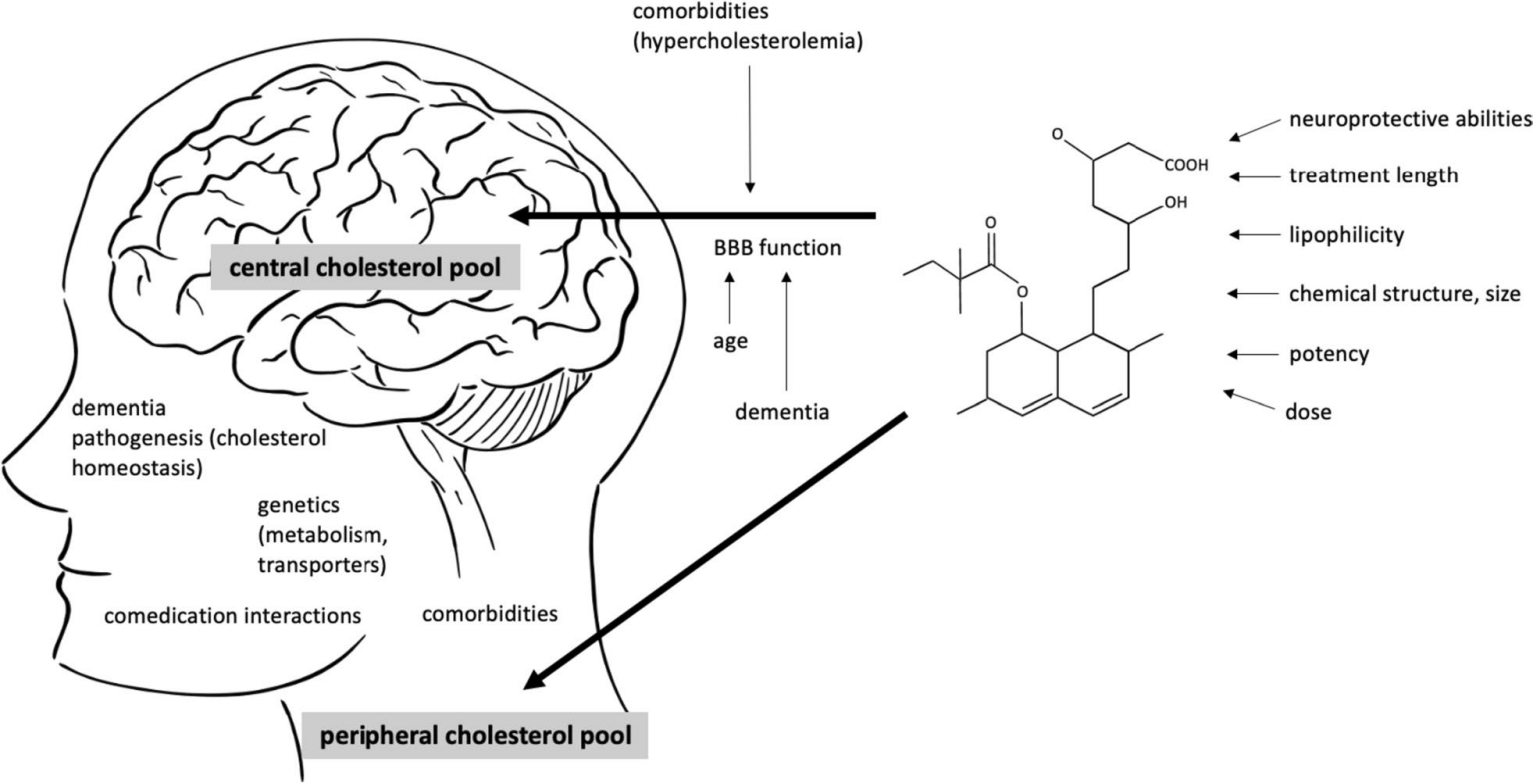
Interaction between the patient’s and medication’s characteristics potentially influence the cognitive effects of statins. Two separate cholesterol pools in the body are thought to be connected to the risk of Alzheimer’s disease (AD), central and peripheral. The brain penetration of statins has been attributed to different factors linked to BBB crossing (lipophilicity of a statin, chemical structure, molecular weight and size of the molecule, different transporters and their genetic polymorphisms). The structure of the barrier itself additionally influences the permeability of statins and is affected by aging, neurodegenerative processes and possibly, peripheral hypercholesterolemia. The overall cognitive effects of statins are likely a result of their central and peripheral actions and are connected to the time of intervention in life and the pathogenesis of AD. Moreover, an interaction of comorbidities and comedication, a sufficient time of treatment and dosages are important. In midlife, protective effect of statins against AD could be achieved through lowering the metabolic risk of hyperlipidaemia. BBB blood–brain barrier, AD Alzheimer’s disease

The overall cognitive effects of statins are likely connected to a complex interaction of factors, related to the patient’s characteristics, integrity of blood–brain barrier permeability [20], characteristics of statins [18], time of treatment, dosages as well as critical time windows in the pathogenesis of dementia [21, 22] (Fig. 1).

Despite the extensive number of observational cohort studies and some clinical trials on statins, their ability to prevent dementia or ameliorate cognitive decline after disease onset is still unclear. A number of mild and reversible short-term cognitive adverse effects [23, 24] contributed to a warning for the labelling of statins by the US Food and Drug Administration. However, numerous large systematic reviews and meta-analyses have not confirmed these adverse cognitive effects [25–29] and some suggested that the use of statins may lower the risk of AD [25, 30–33]. Clinical trials generally reported a null effect [34–36] but were commonly underpowered or used less robust cognitive evaluation tools. Comparably less information is available regarding the effect of statins on cognitive decline in patients with established AD [37–40]. Epidemiological biases inherent to observational design or a heterogeneous design of studies partly explain these discrepancies [41].

The aim of our study was to evaluate the association between statin use and cognitive decline over time in a large cohort of patients diagnosed with AD or mixed AD dementia. We hypothesized that statins that cross the BBB would be associated with less cognitive decline evaluated with mini-mental state examination (MMSE) in these patients.

## Methods

### Study design and registries

We performed a longitudinal cohort study of patients with AD or mixed dementia and indication for lipid-lowering treatment, registered in the Swedish registry for dementia (SveDem). SveDem is a nationwide quality-of-care registry, established in 2007 [42]. All memory clinics and 78 % of primary care centres in Sweden report to SveDem [43]. From this registry, we obtained demographic information (age, sex, living arrangements), date and care unit of registration, type of dementia diagnosis and cognitive status of the patients (MMSE scores) at baseline and follow-ups. The date of the dementia diagnosis in SveDem was set as the index date; 61% of patients had only one entry, 26% had two, 8% had 3 and 5% had more than 3. In total, 80,004 individual patients with dementia were registered in SveDem between 2007 and 2018. All patients were followed until death, emigration or end of follow-up (16 October 2018).

**Fig. 2:**
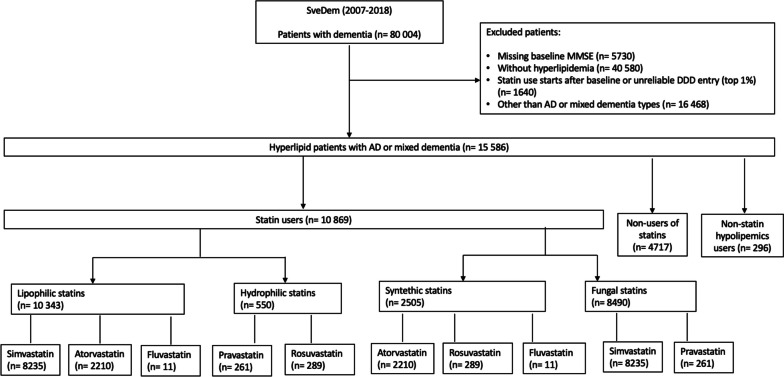
Flowchart of study participants selection. Hyperlipid patients with AD or mixed dementia, registered in SveDem from 2007 to 2018 were included in the study. Among these, we compared cognitive trajectories over time, evaluated with MMSE, in different comparison groups: (1) statin users vs non-users of statins, (2) simvastatin users vs atorvastatin users, (3) simvastatin users vs rosuvastatin users, (4) lipophilic statin users vs hydrophilic statin users, (5) fungal statin users vs synthetic statin users and (6) non-statin lipid-lowering medications users vs statin users

All patients with a missing MMSE score at index date were excluded from the analyses. Only patients diagnosed with hyperlipidaemia (ICD-10 codes from E78.0 to E78.6 obtained from the the Swedish National Patient Registry (NPR), see below) in the preceding 10 years before the index date or those with a prescription of statins (ICD-10 code C10 obtained from the Swedish Prescribed Drug Registry (PDR), see below) in the preceding 6 months before the index date were included in the analyses. Furthermore, the top 1% of statins users sorted by averaged defined daily doses (DDD) were excluded as well, assuming that their consumption data was falsely high and that these individuals bought medication that they did not consume. Figure 2 shows the patient selection flowchart: 15,586 individuals were included for the main analysis.

### Exposures

The exposure drug use was extracted for every SveDem entry. Drug use was defined from the PDR as either the average DDD during the 6-month period preceding each SveDem entry date or simply as a categorical variable (yes/no) during the same period (time-updated exposure). Time-updated exposure means that presence/absence and dose of statins was examined in each 6-month period leading up to each measurement of MMSE (baseline or follow-up). Individual patients starting their statin treatment after the index date were excluded from the analyses because cognitive decline could have affected prescription. All other patients were included in the analyses as non-users. DDD is defined by the World Health Organization as the assumed average maintenance daily dose of a medication for its primary indication in adults [44]. One DDD of simvastatin is equivalent to 30 mg of simvastatin or 20 mg of atorvastatin.

### Medication use

Medication use with their corresponding ATC codes were collected from the PDR that was established in 2005, which includes all prescription medication dispensed at Swedish pharmacies [45]. Lipid-lowering medications included simvastatin, pravastatin, fluvastatin, rosuvastatin, pitavastatin, fibrates, bile acid sequestrants, nicotinic acid and derivates and other non-statin lipid lowering medications. Comedications were calculated as time-updated exposures (yes/no) during the 6-month period preceding the index date. Comedications were selected based on known relevance for patients with dementia and included cardiac drugs, vasoprotectives, platelet aggregation inhibitors, anticoagulants, antipsychotics, anxiolytics, hypnotics, antidepressants, cholinesterase inhibitors, memantine and vitamin D (Appendix). Assumption on the adherence was made based on the collection of the medication at the pharmacy.

### Comorbidities

Comorbidities were obtained with their corresponding ICD-10 codes from the NPR and were coded dichotomously up to 10 years before index date. NPR covers all diagnoses from in-hospital and specialist clinics. Comorbidities were selected based on their known relevance for cognition in patients with dementia and included diabetes mellitus, arrythmia, heart failure, atrial fibrillation, alcohol-related disease, chronic kidney disease, cardiovascular disease, ischemic heart disease, respiratory disease, stroke, anaemia, liver disease, malignancy and obesity (Appendix).

### Covariates

Covariates that were considered included age at baseline, sex (male/female), residency (living with another adult/alone/nursing home/missing), type of dementia diagnostic unit (special memory clinic/primary care centre) and calendar year of dementia diagnosis, all at index date. We selected the covariates that are likely associated with cognitive functions or the probability of receiving statins, based on previous research and/or our clinical knowledge.

The linkage of data from the forementioned registries—SveDem, Swedish National Patient Registry, and Swedish Prescribed Drug Registry—was allowed by the personal identification number of each Swedish citizen. Patient identification was pseudonymized and blinded to the researchers.

### Outcome

The main outcome was cognitive decline, evaluated with MMSE points.

### Statistical analysis

The data were described in terms of mean and standard deviation (SD) for continuous variables and as positive counts (percentages) for categorical variables.

Linear mixed-effects regression models with random intercept and slope were used to investigate the change in MMSE scores over time and to detect differences between statin users and non-users. The model included statin use and time from index date as continuous variables and an interaction between drug use and time. Following our previous work in SveDem [46], a linear trend over time was assumed and the model allowed for a random intercept and random slope for each patient. This model is referred to as the crude model. In an adjusted model, comedications, comorbidities and other covariates were included in the model mostly as categorical variables. Only age, MMSE scores and calendar year at index date were treated as continuous variables. Fully adjusted models included clinical and demographic characteristics (MMSE score at baseline and age at diagnosis, year of diagnosis, sex, residency, comorbidities and comedications). Inverse probability weighting was used to account for the potential effects of general attrition from those lost to follow-up due to dropout. For this purpose, a logistic regression model was fitted to the data to estimate the probability of dropping out within the subsequent year. Dropout was defined as the last observed MMSE score without death or study end occurring in the subsequent year. For more details, see Handels et al. [47].

The analyses were repeated for selected drug groups where drug use was defined as yes/no during the 6-month period before each SveDem entry date and in subgroups defined by gender and age. We split the cohort at the mean age at index date (79.5 years) to create subgroups of younger and older patients. We compared individual statin users. We considered two approaches to divide the statins regarding their functional properties: firstly, lipophilic (simvastatin, atorvastatin, fluvastatin, lovastatin and pitavastatin) or hydrophilic groups (rosuvastatin, pravastatin) [48]. Moreover, we classified them into fungus-derived (simvastatin, pravastatin, lovastatin) or synthetic statins (atorvastatin, cerivastatin, fluvastatin, rosuvastatin, pitavastatin) [15]. Finally, we compared statin users to non-statin lipid-lowering medication users. To evaluate the association between the comparison groups after 3 years, we calculated a theoretical linear extrapolation based on the mixed effect models.

Multiple imputations of MMSE scores [47] and incident users models were two additional models we performed as a sensitivity analysis to check the robustness of our results. The first model deals with bias arising from missing MMSE scores at follow-ups and the second addresses the length of treatment as confounding. Incident users were defined as drug users who did not take out any drug prescription of statins during 12 months before 6-month period preceding each SveDem entry date. Table 1 shows the design of the study.

**Table 1:** Study design

Study design characteristics	Description of characteristics
Population	AD and mixed dementia patients with hyperlipidaemia
Outcome	MMSE points
Statistical model	Linear mixed-effects regression models with inverse probability of drop out weighting
Medication use	Time-updated exposure • Primary analysis: average DDD during the 6-month period preceding index date • Secondary analysis: yes/no use of medication during the 6-month period preceding index date
Analysis	Primary: 1. Statin users vs non-users of statins Secondary: 2. Simvastatin vs atorvastatin users 3. Simvastatin vs rosuvastatin users 4. ^1^Lipophilic vs ^2^hydrophilic statins users 5. ^3^Fungal vs ^4^synthetic statins users 6. Non-statin lipid lowering medication users vs statin users
Covariates	Age, gender, diagnosis year, residency, care unit, comedications, comorbidities
Stratified analyses	1) Gender, 2) age (split at mean age at index date)
Sensitivity analyses	1) Multiple imputations of MMSE, 2) incident users

Patients with AD and mixed dementia who had hyperlipidaemia were included in the study. Outcome was cognitive decline, evaluated with MMSE points. Linear mixed-effects regression models with inverse probability of drop out weighting were used.

Main analysis included comparison of statin users to non-users of statins which included information on average defined daily dose (DDD) of statins in the 6-month period preceding index date.

Secondary analyses consisted of comparisons between simvastatin vs atorvastatin, simvastatin vs rosuvastatin users, lipophilic vs hydrophilic statin users, fungal vs synthetic statin users and non-statin lipid lowering medication users vs statin users, using a dichotomous (yes/no) use of medications in the 6-month period preceding index date.

All analysis was stratified on gender and mean age of the cohort at index date (79.5 years). Sensitivity analysis included multiple imputations of MMSE and incident users.

^1^Lipophilic statins: simvastatin, atorvastatin, fluvastatin

^2^Hydrophilic statins users: pravastatin, rosuvastatin

^3^Fungal statins (type 1): lovastatin, pravastatin, simvastatin

^4^Synthetic statins (type 2): atorvastatin, fluvastatin, rosuvastatin

The cluster robust sandwich estimator was used to estimate standard error of the estimations and two-sided *p*-values were reported. All analyses were conducted using STATA version 16.1 (StataCorp, College Station, TX).

## Results

### Characteristics of study population

As shown in Table 2, our cohort consisted of 15,586 AD or mixed dementia patients with a mean age of 79.5 years (SD = 6.8) at dementia diagnosis. Most patients were women (59.2%). At baseline, all patients scored on average 21 points on MMSE (SD = 5); 10,869 patients (69.7%) used statins in the observation period. The most prescribed statin in the whole cohort was simvastatin (8235, 52.8%) followed by atorvastatin (2210, 14.2%). There were 296 (1.9%) users of a non-statin lipid-lowering medication. Most of the patients resided at home (53.9% with another adult and 40.7% alone) and 5% lived nursing homes. The most common comorbidities in the cohort were hypertension (40.1%), diabetes mellitus (24.4%) and cardiovascular disease (24%).

**Table 2:** Demographic characteristics and comorbidities of the patients with AD and mixed dementia

	**Total cohort (*n* = 15,586)**	**Statin users (*n* = 10,869)**	**Non-users of statins (*n* = 4717)**	***p*-value**
Age at baseline	79.5 (6.8)	78.7 (6.7)	80.7 (6.9)	<0.001
Women	9229 (59.2)	6092 (56.0)	3137 (66.5)	<0.001
Living arrangements				<0.001
With another adult	8395 (53.9)	6230 (57.3)	2165 (45.9)	
Alone	6344 (40.7)	4123 (38.0)	2219 (47.0)	
Nursing home	777 (5.0)	466 (4.3)	311 (6.6)	
Type of dementia diagnostic unit				0.36
Special memory clinic	10,730 (68.8)	7507 (69.1)	3223 (68.3)	
Primary care	4856 (31.2)	3362 (30.9)	1494 (31.7)	
MMSE score at baseline	21.2 (4.8)	21.3 (4.8)	20.8 (4.8)	<0.001
Diabetes mellitus	3797 (24.4)	2922 (26.9)	875 (18.5)	<0.001
Arrhythmia	529 (3.4)	356 (3.3)	173 (3.7)	0.21
Atrial fibrillation	2129 (13.7)	1488 (13.7)	641 (13.6)	0.87
Alcohol-related disorders	198 (1.3)	134 (1.2)	64 (1.4)	0.53
Cardiovascular disease	3739 (24.0)	2904 (26.7)	835 (17.7)	<0.001
Heart failure	1330 (8.5)	940 (8.6)	390 (8.3)	0.43
Myocardial infarction	1921 (12.3)	1480 (13.6)	441 (9.3)	<0.001
Ischemic heart disease	4300 (21.8)	2551 (23.5)	849 (18.0)	<0.001
Respiratory disease	995 (6.4)	681 (6.3)	314 (6.7)	0.36
Haemorrhagic stroke	196 (1.3)	149 (1.4)	47 (1.0)	0.05
Other stroke types	1523 (9.8)	1164 (10.7)	359 (7.6)	<0.001
Anaemia	508 (3.3)	335 (3.1)	173 (3.7)	0.06
Hypertension	6244 (40.1)	4428 (40.7)	1816 (38.5)	0.01
Malignancy	3784 (24.3)	2585 (23.8)	1199 (25.4)	0.03
Liver disease	71 (0.5)	35 (0.3)	26 (0.8)	<0.001
Obesity	179 (1.1)	139 (1.3)	40 (0.8)	0.02
Renal disease	447 (2.9)	323 (3.0)	124 (2.6)	0.24

Data are presented as mean (SD) for continuous variables and *n* (%) for categorical variables. Differences between statin users and non-users were tested with the independent *t*-test for variables age and MMSE score at diagnosis. Otherwise, the chi-squared test was used

The average time of follow-up was 0.86 (SD = 1.40) years and average number of MMSE follow-ups for a patient with measures of MMSE was 1.61 (SD = 0.97). The average decline per year of the cohort was −1.20 points MMSE (95% CI: −1.32; −1.09). The average cognitive decline observed among 6113 patients with at least two MMSE measurements was −2.61 points (SD = 4.57).

Statin users were more commonly male (44 % vs 33.5 %, *p* < 0.001), younger (78.7 vs 80.7 years at baseline, *p* < 0.001) and had a better cognitive status at baseline (21.3 vs 20.8 MMSE points, *p* < 0.001), compared to non-users of statins. As expected, there was a higher prevalence of comorbidities in the former compared to the latter group, such as hypertension, cardiovascular disease, liver disease and diabetes mellitus. Statin users were more likely to be prescribed co-medication, such as antithrombotics, antihypertensives, antidiabetics or psycholeptics. More detailed information is presented in Tables 2 and 3.

**Table 3:** Medication use in patients with AD and mixed dementia

	**Total cohort (*n*= 15,586)**	**Statin users (*n* = 10,869)**	**Non-users of statins (*n* = 4717)**	***p*-value**
Cardiac drugs	2620 (16.8)	1993 (18.3)	627 (13.3)	<0.001
Vasoprotectives	182 (1.2)	108 (1.0)	74 (1.6)	<0.01
Antiplatelets	8324 (53.4)	6444 (59.3)	1880 (39.9)	<0.001
Anticoagulants	2405 (15.4)	1733 (15.9)	672 (14.2)	0.01
Antipsychotics	703 (4.5)	447 (4.1)	256 (5.4)	<0.001
Antidepressants	4736 (30.4)	3261 (30.0)	1475 (31.3)	0.11
Hypnotics	3416 (21.9)	2266 (20.8)	1150 (24.4)	<0.001
Anxiolytics	2064 (13.2)	1365 (12.6)	699 (14.8)	<0.001
Acetylcholinesterase inhibitors	4591 (29.5)	3301 (30.4)	1290 (27.3)	<0.001
Memantine	969 (6.2)	683 (6.3)	286 (6.1)	0.60
Vitamin D	376 (2.4)	248 (2.3)	128 (2.7)	0.11
Diuretics	4294 (27.6)	3136 (28.9)	1158 (24.5)	<0.001
Beta blockers	7248 (46.5)	5481 (50.4)	1767 (37.5)	<0.001
Calcium channel blockers	4347 (27.9)	3257 (30.0)	1090 (23.1)	<0.001
Antihypertensives	12 373 (79.4)	9119 (83.9)	3254 (69.0)	<0.001
Insulin	1599 (10.3)	1247 (11.5)	352 (7.5)	<0.001
Non-insulin antidiabetics	3112 (20.0)	2403 (22.1)	709 (15.0)	<0.001
Non-steroidal anti-inflammatory drugs	1012 (6.5)	705 (6.5)	307 (6.5)	0.96
Bisphosphonate	810 (5.2)	562 (5.2)	248 (5.3)	0.82
Simvastatin	8235 (52.8)	8235 (75.8)	0	N/A
Atorvastatin	2210 (14.2)	2210 (20.3)	0	N/A
Rosuvastatin	289 (1.9)	289 (2.7)	0	N/A
Pravastatin	261 (1.7)	261 (2.4)	0	N/A
Fluvastatin	11 (0.1)	11 (0.1)	0	N/A
Non-statin hypolipemics	296 (1.9)	137 (1.3)	159 (3.4)	<0.001
Fibrate	76 (0.5)	28 (0.3)	48 (1.0)	<0.001
Resin	54 (0.3)	8 (0.1)	46 (1.0)	<0.001
Ezetimibe	170 (1.1)	102 (0.9)	68 (1.4)	0.01

Data are presented as mean (SD) for continuous measures and *n* (%) for categorical measures. Medication groups are classified according to their corresponding ATC codes. Differences between statin users and non-users were tested with a chi-squared test

The sensitivity analysis revealed that a majority of our cohort had used statins at some point in time. Overall, 844 patients were incident users of statins. Compared to incident simvastatin users (*n* = 557), incident atorvastatin users (*n* = 267) had a lower baseline MMSE (20.5 vs 21.4 points, *p* = 0.01) and less commonly received their dementia diagnosis at special memory clinic (60.3 % vs 78.8 %, *p* < 0.001). (Supplementary table 7).

### Cognitive decline in different treatment groups

#### Statin users compared to non- users of statins

**Fig. 3:**
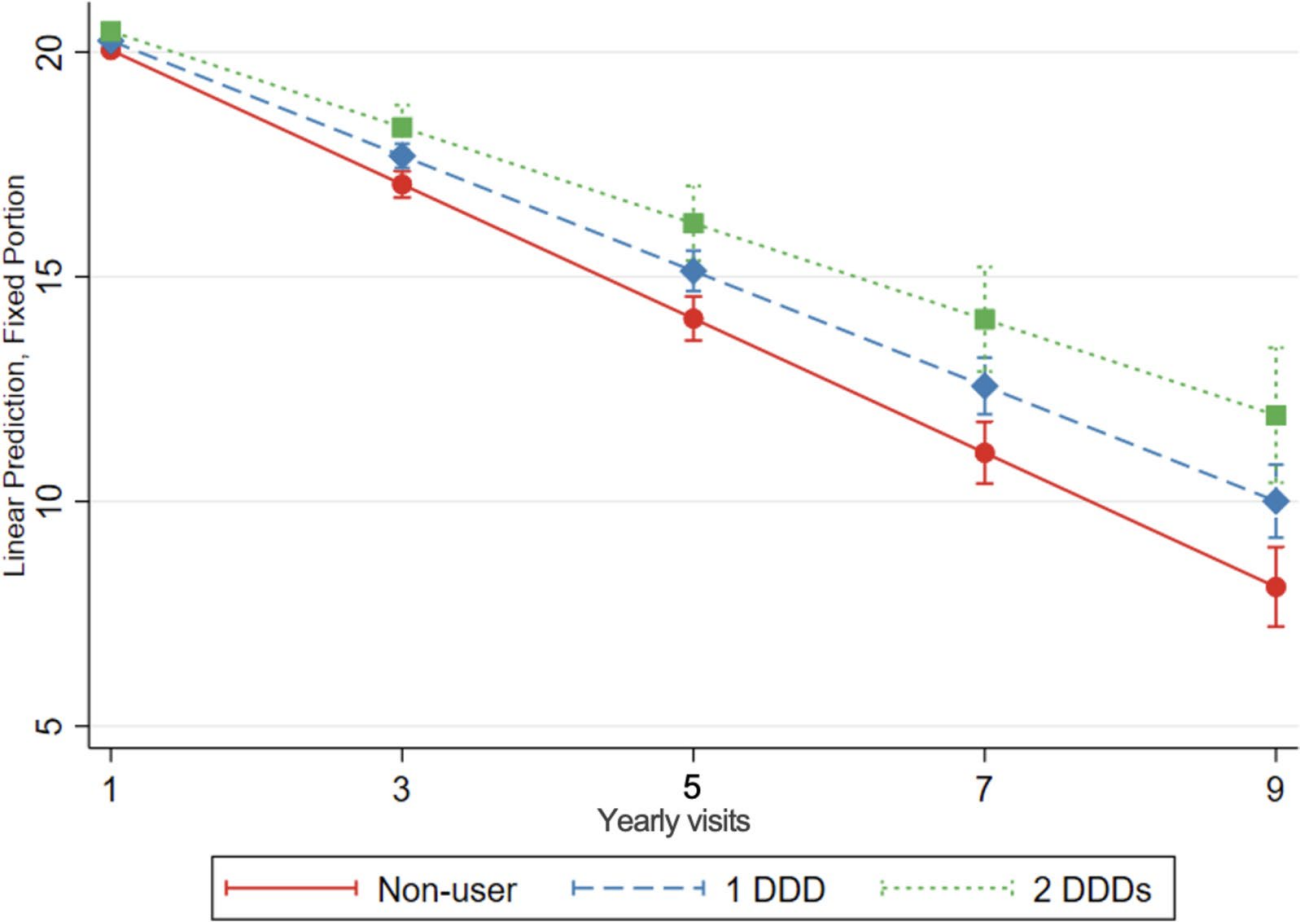
Cognitive decline, evaluated with change in MMSE score over time, in statin users compared to non-users of statins. The graph shows the association between increasing doses of statin treatment and MMSE over time, as predicted from the model. Linear mixed-effects regression model, adjusted for demographic characteristics, comorbidities and comedication, with inverse probability weighting. DDD defined daily dose. DDD is defined by the World Health Organization as the assumed average maintenance daily dose of a medication for its primary indication in adults. Yearly visit 1 represent the first MMSE measurement (baseline)

Statin use was associated with a slower cognitive decline over time compared to no use of statins. After taking an average of 1 DDD of statins for a year, statin users had 0.21 more MMSE points (95% CI: 0.12; 0.32) compared to non-users. There was a dose-response effect. After 3 years of taking an average 1 DDD of statins, statin-treated patients had 0.63 points more MMSE points (95% CI: 0.33; 0.94) (Table 4, Fig. 3). These results were consistent in subgroup analysis and when considering imputed missing MMSE values and analysis restricted to incident users (Supplementary table 6) and in subgroup analyses (Supplementary table 1). We conducted post hoc analyses stratifying by dementia type (Alzheimer or mixed dementia; results not shown) with similar results to those presented for the whole group.

**Table 4:** Cognitive trajectories in different treatment groups

	Crude model			Adjusted model		
	Coeff.	95% CI	*p*-value	Coeff.	95% CI	*p*-value
Statin users (ref. non-users of statins)						
Average	0.05	-0.12; 0.21	0.596	0.01	-0.02; 0.01	0.670
Per year	0.15	0.04; 0.26	0.007	0.21	0.12; 0.32	<0.001
After 3 years	0.50	0.21; 0.80	0.001	0.63	0.33; 0.94	<0.001
Simvastatin users (ref.- atorvastatin users)						
Average	-0.22	-0.48; 0.03	0.090	-0.03	-0.07; 0.01	0.105
Per year	0.30	0.07; 0.53	0.010	0.35	0.03; 0.67	0.035
After 3 years	0.68	0.02; 1.34	0.043	1.01	0.06; 1.97	0.038
Simvastatin users (ref. rosuvastatin users)						
Average	-0.48	-1.11; 0.14	0.131	-0.03	-0.09; 0.03	0.349
Per year	0.48	0.08; 0.87	0.018	0.35	0.09; 0.61	0.008
After 3 years	0.95	-0.12; 2.01	0.081	1.03	0.26; 1.80	0.009
Lipophilic statin users (ref.- hydrophilic statin users)						
Average	-0.04	-0.55; 0.48	0.883	0.01	-0.04; 0.04	0.953
Per year	0.03	-0.41; 0.47	0.902	0.08	-0.25; 0.41	0.635
After 3 years	0.04	-1.19; 1.28	0.944	0.24	-0.75; 1.24	0.632
Fungal statin users (ref. synthetic statin users)						
Average	-0.38	-0.64; -0.11	0.006	-0.03	-0.07; 0.01	0.067
Per year	0.24	0.02; 0.46	0.035	0.28	-0.02; 0.59	0.071
After 3 years	0.34	-0.30; 0.98	0.301	0.82	-0.09; 1.72	0.079
Non-statin lipid-lowering medication users (ref. statin users)						
Average	0.07	-0.78; 0.93	0.866	-0.006	-0.74; 0.09	0.889
Per year	0.74	0.90; -2.37	0.896	0.40	-1.33; 2.13	0.654
After 3 years	2.28	-2.34; 6.90	0.333	1.19	-3.35; 6.34	0.649

Linear mixed-effects regression model with inverse probability weighting, crude model and adjusted model for selected demographic characteristics, comorbidities and comedication (details in methods). Coefficient for the point-wise difference in MMSE points over time between comparison groups, 95% CI and two-sided *p*-values are reported. Average refers to the average difference in MMSE points between comparison groups at any time point

#### Simvastatin users compared to atorvastatin users

**Fig. 4:**
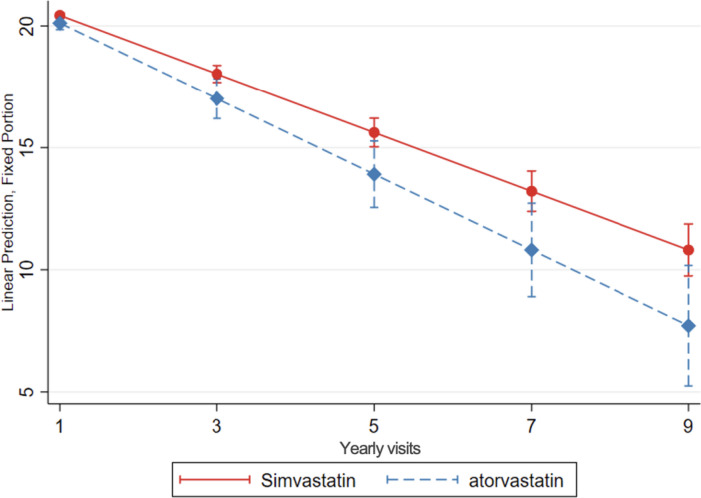
Cognitive decline, evaluated with change in MMSE score over time, in simvastatin compared to atorvastatin users. Linear mixed-effects regression model, adjusted for demographic characteristics, comorbidities and comedication, with inverse probability weighting. Yearly visit 1 represent the first MMSE measurement (baseline)

Simvastatin users exhibited a slower cognitive decline over time, compared to atorvastatin users (0.35 more MMSE points per year of follow-up, 95% CI: 0.03; 0.67 and 1.01 more MMSE points after 3 years, 95% CI: 0.06; 1.97) (Table 4, Fig. 4).

When stratifying analyses for gender and age, the protective association for cognition of simvastatin compared to atorvastatin, was only statistically significant in the participants aged <79.5 years (which was the mean age of the total sample). Younger users of simvastatin had a slower decline of MMSE (0.28 points more per year, 95% CI: 0.03; 0.54, and 0.80 points more after 3 years, 95% CI: 0.05; 1.55) compared to younger atorvastatin users (Table 5).

**Table 5:** Cognitive decline in subgroups of simvastatin users compared to atorvastatin users

Simvastatin users (ref. atorvastatin users)												
	Men			Women			Younger			Older		
	Coeff.	95% CI	*p*-value	Coeff.	95% CI	*p*-value	Coeff.	95% CI	*p*-value	Coeff.	95% CI	*p*-value
Average	-0.03	-0.09; 0.02	0.200	-0.03	-0.08; 0.02	0.246	-0.05	-0.11; 0.01	0.123	-0.02	-0.06; 0.02	0.369
Per year	0.49	-0.07; 1.04	0.086	0.23	-0.09; 0.56	0.153	0.28	0.03; 0.54	0.029	0.60	-0.43; 1.63	0.251
After 3 years	1.42	-0.22; 3.07	0.089	0.67	-0.28; 1.63	0.168	0.80	0.05; 1.55	0.037	1.79	-1.27; 4.85	0.252

Linear mixed-effects regression model with inverse probability weighting, adjusted model for selected demographic characteristics, comorbidities and comedication (details in methods). Analysis stratified on sex and mean age (79.5 years) at index date. Coefficient for the point-wise difference in MMSE points over time between simvastatin and atorvastatin users, 95 % CI and two-sided *p*-values are reported. Average refers to the average difference in MMSE points between comparison groups at any time point

This protective association was statistically significant in multiple imputations of MMSE model but not in incident users (0.56 less MMSE points per year, 95% CI: -1.13; 0.01) (Supplementary table 6). We conducted post hoc analyses stratifying by dementia type (Alzheimer or mixed dementia): in mixed dementia, simvastatin use was associated with 1.57 more MMSE points MMSE at 3 years (95% CI 0.79; 2.34) while results were not significant for the Alzheimer group (0.49 more points MMSE at 3 years; 95% CI -0.76; 1.76).

#### Simvastatin users compared to rosuvastatin users

**Fig. 5:**
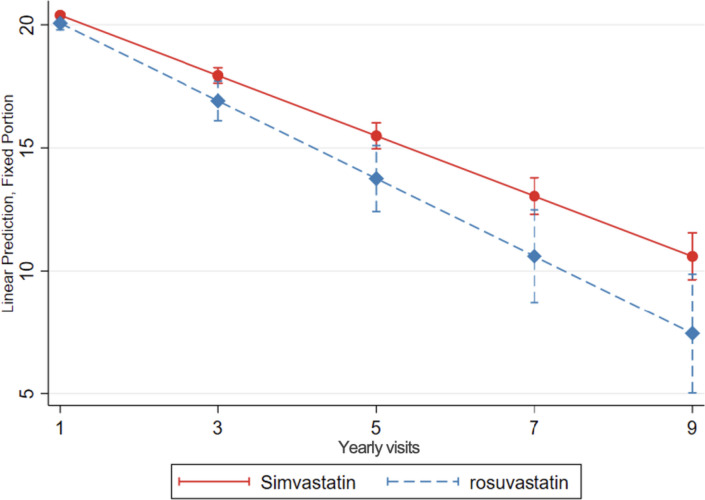
Cognitive decline, evaluated with change in MMSE scores over time, in simvastatin compared to rosuvastatin users. Linear mixed-effects regression model, adjusted for demographic characteristics, comorbidities, and co-medication, with inverse probability weighting. Yearly visit 1 represent the first MMSE measurement (baseline)

Simvastatin users had a slower MMSE decline, compared to rosuvastatin users (0.35 more MMSE points per year of follow-up, 95% CI: 0.09; 0.61, and 1.03 more MMSE points after 3 years, 95% CI: 0.26; 1.80) (Table 4, Fig. 5).

In subgroup analysis, these results remained statistically significant in women and younger patients (Supplementary table 2).

The associations remained protective when imputing missing MMSE values. However, incident simvastatin users had a faster decline of MMSE compared to incident rosuvastatin users (1.63 less MMSE points per year of follow-up, 95% CI: -3.18; -0.07 and 4.77 less MMSE points after 3 years, 95% CI: -9.46; -0.07) (Supplementary table 6).

#### Lipophilic statin users compared to hydrophilic statin users

We did not find significant differences in MMSE decline in lipophilic statin users (simvastatin, atorvastatin, fluvastatin users) (Table 4) or when considering imputed values of missing MMSE, compared to hydrophilic statins users (rosuvastatin, pravastatin users). However, it was faster in incident users of lipophilic statins (1.32 less MMSE points per year, 95% CI: -2.46; -0.18), and 3.84 less points after 3 years, 95% CI: -7.28; -0.41), compared to hydrophilic statins (Supplementary table 6). These analyses were not statistically significant in sub-analysis of age groups and sex (Supplementary table 3).

#### Fungal statin users compared to synthetic statin users

Use of fungal statins (simvastatin, pravastatin users) was not associated with a difference in MMSE decline compared to synthetic statin users (atorvastatin, rosuvastatin, fluvastatin users) (Table 4). In a subgroup analysis, the MMSE decline was slower in younger fungal statin users (0.26 more points per year, 95% CI: 0.03; 0.49, and 0.73 more points after 3 years, 95% CI: 0.05; 1.42) (Supplementary table 4). However, the decline was faster when analysing only incident fungal users, compared to incident synthetic users (0.61 points less per year, 95% CI: -1.19; -0.04, and 1.83 points less after 3 years, 95% CI: -3.55; -0.12) (Supplementary table 6).

#### Non-statin lipid-lowering medication users compared to statin users

We did not observe a significant difference in MMSE decline between non-statin lipid lowering medication users, compared to statin users (Table 4).

In some subgroup analyses, users of non-statin lipid lowering medication had a slower MMSE decline (Supplementary table 5).

## Discussion

In this longitudinal Swedish registries-based observational study of patients with AD or mixed AD dementia, we discovered a dose-dependent cognitive benefit over time in statin users compared to non-users of statins. Additionally, we discovered a slower MMSE decline over time in patients taking simvastatin, compared to either atorvastatin or rosuvastatin users. Younger users of simvastatin had a slower MMSE decline compared to younger atorvastatin users. We did not observe a difference in MMSE decline depending on lipophilicity. Incident users’ analysis revealed inconsistent findings which could be potentially explained with time-dependant non-linear association between effect of statins on cognitive processes or through differences and selection of these users.

### Different statins

Simvastatin was the most used statin in Sweden when our data was collected. Accordingly, this makes comparisons among different statins or groups difficult, often lacking enough power. A beneficial role of simvastatin in early dementia is biologically plausible, when there are high levels of neuroinflammation as this lipophilic statin readily crosses the BBB and could exert various neuroprotective properties, such as protection against tau hyperphosphorylation and mediation of brain cholesterol homeostasis [18]. Research on animal models of AD further support the beneficial effects of simvastatin on cognition through different mechanisms [49, 50]. Clinical trials in patients with mild to moderate AD reported a neutral [35] or a beneficial effect of simvastatin [51], using MMSE as a cognitive outcome. Findings were limited by relatively short trial duration and low number of participants and were therefore possibly underpowered. To our best knowledge, our study is the first observational study to compare cognitive decline between different statins in patients with established AD and mixed dementia. A careful adjustment for comedication in general and cholinesterase inhibitors in particular is important, as our group previously discovered a small long-term beneficial effect on cognition in AD and mixed dementia patients treated with cholinesterase inhibitors [46].

In our study, the analysis including only incident users showed an opposite association. A possible explanation to inconsistent results of incident user design may be related to a temporally dependent biphasic effect of statin therapy on cholesterol metabolites, as shown in a study which included asymptomatic patients at risk of AD [52]. In this study, statins initially reduced cholesterol metabolites in the cerebrospinal fluid, reaching a nadir at 6–7 months, followed by a return to baseline and an overshoot at two years. Moreover, several differences between incident users compared to all users exist which could partly contribute to the discrepancies. The individual characteristics of incident users, such as baseline MMSE differences, or a possible selection of these smaller groups of patients through individual physicians’ preferences, might have influenced their cognitive trajectories which could not be accounted for in adjusted models.

### Lipophilicity and chemical characteristics (fungal or synthetic statins)

Several biochemical characteristics of statins probably influence the functional effects of statins on cholesterol metabolism and cognition. Statins with a higher lipophilicity (e.g., simvastatin, atorvastatin, fluvastatin) may cross the BBB more easily compared to more hydrophilic statins (rosuvastatin, pravastatin) [18]. Additionally, the size and orientation of a statin molecule may influence the BBB permeability of statins which explained a low ability of a lipophilic atorvastatin to cross the BBB, due to its large size [18]. In our study, we did not observe a difference in cognitive decline when comparing users of lipophilic to hydrophilic statins in most models and subgroup analyses. However, MMSE decline was faster in incident users of lipophilic statins. Due to forementioned Swedish prescription patterns, the comparisons in our study were driven by simvastatin and atorvastatin users compared to rosuvastatin users. To the best of our knowledge, we are not aware of another observational study which compared cognitive decline between lipophilic and hydrophilic statin users in AD patients.

Fungal statins (simvastatin and pravastatin) differ from the synthetic statins (atorvastatin, rosuvastatin, fluvastatin) in several functional characteristics. Synthetic statins were shown to form more interactions which leads to a stronger inhibition of HMG-CoA reductase and a higher potency [53]. Moreover, fungus-derived statins were observed to have a high permeability through the blood–brain barrier and cause a reduction of cholesterol levels as well as lower a burden of neurofibrillary tangles in animal models [54]. In our study, this comparison between fungal and synthetic statins was driven by simvastatin and atorvastatin users, so this classification did not add further information. To the best of our knowledge, no previous studies compared cognitive decline in AD or mixed dementia patients among fungal and synthetic statins. A recent cohort study comparing different incident statin users found a higher risk of AD in fungal statins compared to synthetic, as well as higher risk in lipophilic statins compared to hydrophilic statins; however, the risk was reduced in sensitivity analysis [55].

### Non-statin lipid-lowering medications

The confidence intervals for this comparison were broad and did not reach statistical significance. However, MMSE decline was slower in some subgroups of non-statin lipid-lowering medications (men and younger users). Statins and other hypolipemics, such as gemfibrozil, represent another interesting comparison group since they are both prescribed for hyperlipidaemia, therefore diminishing indication bias, and could exhibit cognitive effects through different metabolic pathways. Gemfibrozil attenuated amyloid burden as well as neuroinflammation and improved the memory in AD mouse models through activation of PPAR-alpha in a recent study [56], but our study was probably underpowered for this comparison.

### Statin dose, potency, treatment length and time window for intervention

The dose, potency or duration of treatment have been recognized as important factors when evaluating the effects of statins on cognition. Most work has been done on evaluating these factors in the prevention of dementia or AD [32, 33, 57]. A dose-response was observed in a large cohort study which included only AD patients [58]. In our study, a dose-effect was observed when comparing statin users and non-users. The prediction model showed a benefit after 3 years, which is an estimated brain cholesterol turnover rate in adults, but most of the data in our cohort aggregates towards earlier follow-ups. A time window of intervention with statins regarding the neuropathogenesis of dementia, or life course of a patient, might exist as the neurodegenerative pathological changes of AD begin decades prior to clinical symptoms [59]. The protective effect of statins could be achieved in a long-term amelioration of brain vascular burden, restoration of disturbed central cholesterol homeostasis and neuroprotective effects, possibly in preclinical [60] or early stages of AD [61, 62].

### Statin use compared to no use of statins

An extensive evaluation of the possible role of statins in preventing dementia has been performed in the last two decades [25, 26, 32, 33, 41, 63–65] but comparably less studies included patients with already established AD [25, 31, 38–41, 64]. Clinical trials of statin use in AD patients did not report a clear benefit to ameliorate cognitive decline [38, 40, 64]. Observational cohort studies of patients with AD and a various follow-up ranging from 10 months to over 10 years, reported a slower [61, 66–68] or similar [69] cognitive decline in statins users compared to non-users. Findings from our analysis comparing statin use to no use which imply a possible dose-dependent beneficial role of statins in patients with AD is in accord with many of these previous studies. However, comparing statin users and no-users introduces several important biases.

Importantly, hyperlipidaemia is an indication for statin use in midlife and represents a risk factor for dementia and AD. On the other hand, low cholesterol level in late life has been recognized as a measure of frailty or prodromal stage of dementia, particularly AD [70]. These facts can lead an indication bias when comparing users to non-users. Secondly, clinicians might be less likely to prescribe statins to older patients, especially those with pre-existing cognitive decline, frailty, or comorbidities since the risk of possible side effects and diminished life expectancy outweighs the benefit of medication. Furthermore, cognitive impairment could lead to a discontinuation of statins or drop-out from study, which would lead to a false beneficial association [41, 71]. Older patients who receive statins for their hypercholesterolemia could naturally possess a lower risk of dementia or reflect a better cognitive trajectory [72], leading to reverse causation.

### Strengths and limitations

Our study has several strengths and limitations. We can report associations but are not able to draw the conclusions on causality. This study was meant as an exploratory analysis which requires confirmation. We considered a variety of comorbidities and comedications in our models; however, a few important covariates were not available for our analysis, such as cholesterol levels, *ApoE* status and possible genetic polymorphisms specific for different populations [73]. However, we addressed this issue with a selection of a population with indication for treatment and used multivariate adjusting to balance the differences between the groups as well as performed several sensitivity analyses. Patient adherence to medication was indirectly assumed based on the dispensation of medication at pharmacies. There was a considerable number of drop-out participants and missing values on MMSE. MMSE was the only measure of cognitive decline in our study and is less robust to detect cognitive changes in different cognitive domains. We chose to only include observed MMSE scores in the analyses to limit the risk of creating false data with imputation but there is, of course, a risk of selection bias. We restricted the study population to those individuals with drug use at index date to ensure that we can follow a statin user’s cognitive decline from the beginning. The results from the sensitivity analyses considering multiple imputations of MMSE are in line with our main findings and confirmed our choices. However, the results of analysis on incident users were not consistent. Another important strength of our study lies in carefully selected statistical methods. Use of linear mixed modelling with multiple imputation is currently regarded as a superior method to account for the attrition bias [72]. We considered a reasonably long follow-up and performed a large, population-based study. We examined the use of statins before dementia to explore the reverse causality of cognition influencing the adherence or use of statin but cannot completely exclude this problem with our current methods.

## Conclusions

Our population-based exploratory cohort study of patients with AD or mixed dementia adds to a growing body of evidence that statins are not detrimental for cognition. Moreover, statins might exhibit a long-term cognitive benefit these patients who have indication for lipid-lowering treatment. However, our findings warrant a confirmational study. We believe that our findings should further encourage clinicians to select eligible patients with dementia to benefit from prevention of their cardiovascular and cerebrovascular disease with statins. Further research of the pathogenesis of dementia is warranted. Acknowledging dementia as a complex, multifactorial syndrome where different pathogenic processes and risk factors are at play at different stages of dementia, it would be plausible to examine the combined effects of several medications affecting different metabolic pathways in well-defined subtypes of dementia. Moreover, the role of lipid metabolism dysregulation to the pathogenesis of AD should be further explored, taking the genetic factors into consideration. Further research is needed to decipher the non-consistent results in incident statin users, where time since prescription may be an important factor.

### Supplementary Information

**Additional file 1:** **Supplementary table 1.** Cognitive decline in subgroups of statins users vs users of non-users of statins. **Supplementary table 2.** Cognitive decline in subgroups of simvastatin users vs rosuvastatin users. **Supplementary table 3.** Cognitive decline in subgroups of lipophilic statins users vs hydrophilic statins users. **Supplementary table 4.** Cognitive decline in subgroups of fungal statins users vs synthetic statins users. **Supplementary table 5.** Cognitive decline in subgroups of non-statin lipid-lowering medication users vs statins users. **Supplementary table 6.** Cognitive decline in different treatment groups, sensitivity analysis. **Supplementary table 7.** Characteristics of incident users.

## Data Availability

Following the Swedish and EU legislation, the data are not available for public access. In order to obtain the data from Swedish registries, researches must apply to the steering committees of the registries as well as relevant government authorities, after obtaining the ethical approval.

## Appendix

### ICD-10 codes for comorbidities

Diabetes mellitus/insulin/other antidiabetics (E1[0-4] A10A A10B), arrhythmia (I49), alcohol-related disease (E244 F10 G312 G621 G721 I426 K292 K70 K860 O354 P043 Q860 T51 Y90 Y91 Z502 Z714), chronic kidney disease (N18 N19 I120 I131 N032 N033 N034 N035 N036 N037 N052 N053 N054 N055 N056 N057 N18 N19 N250 T856 T857 Z490 Z491 Z492 Z940 Z992), heart failure (I500, I501, I509), cardiovascular disease (I21[0-4] I219 I220 I221 I228 I229 I25 I63 I739 I70), myocardial infarction (I21 I22 I252), respiratory disease (J4[0-7] J6[0-7] J684 J701 J703), haemorrhagic stroke (I60 I61 I62), other stroke (I63 I64 I69), anaemia (D50 D62), liver failure (K7), ischemic heart disease (I20 I21 I22 I23 I24 I25), malignancy (C[0-9][0-9] D[1-3]D4[0-8]) and obesity (E66).

### ATC codes for lipid-lowering medication

Simvastatin (C10AA01 C10BA02 C10BX01 C10BX04), atorvastatin (C10AA05 C10BA05 C10BX03, C10BX06 C10BX08 C10BX11 C10BX12 C10BX15), pravastatin (C10AA03), fluvastatin (C10AA04), rosuvastatin (C10AA07 C10BA06 C10BX05 C10BX07 C10BX09 C10BX10 C10BX13 C10BX14), pitavastatin (C10AA08), fibrates (C10AB), bile acid sequestrants (C10AC), nicotinic acid and derivates (C10AD) and other lipid modifying agents (C10AX).

### ATC codes for comedication

Cardiac drugs (C01), vasoprotectives (C05), platelet aggregation inhibitors (B01AC), anticoagulants (B01AA B01AB B01AF B01AE07), antipsychotics (N05A), anxiolytics (N05B), hypnotics (N05C), antidepressants (N06A), anticholinesterases (N06DA), memantine (N06DX01) and vitamin D (A11CC).

## Abbreviations

AD: Alzheimer’s disease
MMSE: Mini-mental state examination
SveDem: Swedish Registry for Cognitive/Dementia Disorders
Aβ: Amyloid beta
ApoE4: Apolipoprotein E4
HMG-CoA reductase: 3-Hydroxy-3-methylglutaryl coenzyme A reductase
BBB: Blood–brain barrier
NPR: National Patient Registry
PDR: Swedish Prescribed Drug Registry
DDD: Defined daily dose
ICD-10: International Classification of Diseases, 10th Revision
SD: Standard deviation
CI: Confidence interval
