# Supplementary material for: Statins and cognitive decline in patients with Alzheimer’s and mixed dementia: a longitudinal registry-based cohort study
Source: Alzheimers Res Ther. 2023 Dec 20;15:220. doi: 10.1186/s13195-023-01360-0 (PMC10731754; doi:10.1186/s13195-023-01360-0)
Supplement: Supplementary file 1 — Additional file 1: Supplementary table 1. Cognitive decline in subgroups of statins users vs users of non-users of statins. Supplementary table 2. Cognitive decline in subgroups of simvastatin users vs rosuvastatin users. Supplementary table 3. Cognitive decline in subgroups of lipophilic statins users vs hydrophilic statins users. Supplementary table 4. Cognitive decline in subgroups of fungal statins users vs synthetic statins users. Supplementary table 5. Cognitive decline in subgroups of non-statin lipid-lowering medication users vs statins users. Supplementary table 6. Cognitive decline in different treatment groups, sensitivity analysis. Supplementary table 7. Characteristics of incident users. [file 13195_2023_1360_MOESM1_ESM.docx]

**Supplementary table 1** Cognitive decline in subgroups of statins users vs users of non-users of statins

| **Statin users (ref.- non-users of statins)** | | | | | | | | | | | | |
| --- | --- | --- | --- | --- | --- | --- | --- | --- | --- | --- | --- | --- |
|  | **Men** | | | **Women** | | | **Younger** | | | **Older** | | |
|  | Coeff. | 95% CI | p-value | Coeff. | 95% CI | p-value | Coeff. | 95% CI | p-value | Coeff. | 95% CI | p-value |
| Average | 0.01 | -0.04; 0.02 | 0.535 | 0.0003 | -0.02; 0.02 | 0.975 | 0.002 | -0.03; 0.03 | 0.881 | -0.006 | -0.03; 0.01 | 0.533 |
| Per year | 0.25 | 0.09; 0.41 | 0.002 | 0.18 | 0.05; 0.31 | 0.008 | 0.17 | 0.05; 0.29 | 0.006 | 0.32 | 0.13; 0.52 | 0.001 |
| After three years | 0.74 | 0.26; 1.21 | 0.002 | 0.53 | 0.14; 0.92 | 0.007 | 0.51 | 0.16; 0.86 | 0.005 | 0.96 | 0.38; 1.54 | 0.001 |

Linear mixed-effects regression model with inverse probability weighting, adjusted model for selected demographic characteristics, comorbidities and comedication (details in methods). Analysis stratified on sex and mean age (79.5 years) at index date. Coefficient for the point-wise difference in MMSE points over time between statin users and non-users of statins, 95% CI and two-sided p-values are reported.

**Supplementary table 2** Cognitive decline in subgroups of simvastatin users vs rosuvastatin users

| **Simvastatin users (ref.- rosuvastatin users)** | | | | | | | | | | | | |
| --- | --- | --- | --- | --- | --- | --- | --- | --- | --- | --- | --- | --- |
|  | **Men** | | | **Women** | | | **Younger** | | | **Older** | | |
|  | Coeff. | 95% CI | p-value | Coeff. | 95% CI | p-value | Coeff. | 95% CI | p-value | Coeff. | 95% CI | p-value |
| Average | -0.06 | -1.45; 0.03 | 0.221 | -0.02 | -0.10; 0.06 | 0.638 | -0.04 | -0.13; 0.06 | 0.479 | -0.01 | -0.07; 0.05 | 0.744 |
| Per year | 0.15 | -0.10; 1.32 | 0.234 | 0.85 | 0.35; 1.34 | 0.001 | 0.44 | 0.14; 0.74 | 0.004 | -0.42 | -1.31; 0.47 | 0.351 |
| After three years | 0.41 | -0.37; 1.18 | 0.302 | 2.52 | 1.06; 3.99 | 0.001 | 1.29 | 0.39; 2.18 | 0.005 | -1.28 | -3.93; 1.38 | 0.346 |

Linear mixed-effects regression model with inverse probability weighting, adjusted model for selected demographic characteristics, comorbidities and comedication (details in methods). Analysis stratified on sex and mean age (79.5 years) at index date. Coefficient for the point-wise difference in MMSE points over time between simvastatin and rosuvastatin users, 95 % CI and two-sided p-values are reported.

**Supplementary table 3** Cognitive decline in subgroups of lipophilic statins users vs hydrophilic statins users

| **Lipophilic statins users (ref.- hydrophilic statins users)** | | | | | | | | | | | | |
| --- | --- | --- | --- | --- | --- | --- | --- | --- | --- | --- | --- | --- |
|  | **Men** | | | **Women** | | | **Younger** | | | **Older** | | |
|  | Coeff. | 95% CI | p-value | Coeff. | 95% CI | p-value | Coeff. | 95% CI | p-value | Coeff. | 95% CI | p-value |
| Average | 0.02 | -0.10; 0.05 | 0.488 | 0.02 | -0.03; 0.75 | 0.449 | 0.006 | -0.07; 0.08 | 0.869 | 0.0006 | -0.05; 0.05 | 0.978 |
| Per year | 0.18 | -0.10; 0.46 | 0.210 | -0.05 | -0.76; 0.65 | 0.881 | 0.15 | -0.20; 0.50 | 0.395 | -0.24 | -0.76; 0.29 | 0.374 |
| After three years | 0.51 | -0.32; 1.34 | 0.228 | -0.14 | -2.25; 1.97 | 0.896 | 0.46 | -0.58; 1.51 | 0.387 | -0.71 | -2.26; 0.85 | 0.371 |

Linear mixed-effects regression model with inverse probability weighting, adjusted model for selected demographic characteristics, comorbidities and comedication (details in methods). Analysis stratified on sex and mean age (79.5 years) at index date. Coefficient for the point-wise difference in MMSE points over time between lipophilic and hydrophilic statins users, 95 % CI and two-sided p-values are reported.

**Supplementary table 4** Cognitive decline in subgroups of fungal statins users vs synthetic statins users

| **Fungal statins users (ref.- synthetic statins users)** | | | | | | | | | | | | |
| --- | --- | --- | --- | --- | --- | --- | --- | --- | --- | --- | --- | --- |
|  | **Men** | | | **Women** | | | **Younger** | | | **Older** | | |
|  | Coeff. | 95% CI | p-value | Coeff. | 95% CI | p-value | Coeff. | 95% CI | p-value | Coeff. | 95% CI | p-value |
| Average | -0.04 | -0.09; 0.01 | 0.149 | -0.03 | -0.08; 0.02 | 0.191 | -0.05 | -0.10; 0.01 | 0.121 | -0.02 | -0.07; 0.02 | 0.264 |
| Per year | 0.44 | -0.04; 0.93 | 0.074 | 0.13 | -0.21; 0.48 | 0.444 | 0.26 | 0.03; 0.49 | 0.028 | 0.39 | -0.78; 1.56 | 0.510 |
| After three years | 1.29 | -0.14; 2.73 | 0.078 | 0.37 | -0.65; 1.39 | 0.478 | 0.73 | 0.05; 1.42 | 0.036 | 1.16 | -2.33; 4.64 | 0.515 |

Linear mixed-effects regression model with inverse probability weighting, adjusted model for selected demographic characteristics, comorbidities and comedication (details in methods). Analysis stratified on sex and mean age (79.5 years) at index date. Coefficient for the point-wise difference in MMSE points over time between fungal and synthetic statins users, 95% CI and two-sided p-values are reported.

**Supplementary table 5** Cognitive decline in subgroups of non-statin lipid-lowering medication users vs statins users

| **Non-statin lipid-lowering agents users (ref- statin users)** | | | | | | | | | | | | |
| --- | --- | --- | --- | --- | --- | --- | --- | --- | --- | --- | --- | --- |
|  | **Men** | | | **Women** | | | **Younger** | | | **Older** | | |
|  | Coeff. | 95% CI | p-value | Coeff. | 95% CI | p-value | Coeff. | 95% CI | p-value | Coeff. | 95% CI | p-value |
| Average | -0.07 | -0.15; 0.01 | 0.092 | 0.07 | -0.04; 0.18 | 0.237 | 0.01 | -0.09; 0.12 | 0.793 | 0.02 | -0.10; 0.15 | 0.709 |
| Per year | 1.37 | 0.52; 2.22 | 0.002 | 1.37 | -4.72; 1.98 | 0.422 | 0.96 | 0.07¸1.85 | 0.034 | -3.07 | -9.71; 3.58 | 0.366 |
| After three years | 4.04 | 1.51;6.57 | 0.002 | 4.04 | -14.01; 5.93 | 0.427 | 2.89 | 0.25; 5.53 | 0.032 | -9.18 | -29.02; 10.67 | 0.365 |

Linear mixed-effects regression model with inverse probability weighting, adjusted model for selected demographic characteristics, comorbidities and comedication (details in methods). Analysis stratified on sex and mean age (79.5 years) at index date. Coefficient for the point-wise difference in MMSE points over time between non-statin lipid-lowering medication users and statins users, 95% CI and two-sided p-values are reported.

**Supplementary table 6** Cognitive decline in different treatment groups, sensitivity analysis

| **Statin users (ref.- non-users of statins)** | | | | | | |
| --- | --- | --- | --- | --- | --- | --- |
|  | **Multiple imputations model** | | | **Incident users** | | |
|  | Coeff. | CI | p-value | Coeff. | CI | p-value |
| Average | -0.01 | -0.02; 0.01 | 0.390 | -0.02 | -0.07; 0.03 | 0.401 |
| Per year | 0.21 | 0.12; 0.31 | <0.001 | 0.44 | 0.11; 0.77 | 0.008 |
| After three years | 0.64 | 0.35; 0.92 | <0.001 | 1.31 | 0.31; 2.31 | 0.010 |
| **Simvastatin users (ref.- atorvastatin users)** | | | | | | |
|  | **Multiple imputations model** | | | **Incident users** | | |
|  | Coeff. | CI | p-value | Coeff. | CI | p-value |
| Average | -0.02 | -0.04; 0.01 | 0.238 | 0.004 | -0.08; 0.09 | 0.923 |
| Per year | 0.32 | 0.03; 0.62 | 0.031 | -0.56 | -1.13; 0.01 | 0.055 |
| After three years | 0.95 | 0.08; 1.82 | 0.032 | -1.17 | -3.38; 0.04 | 0.055 |
| **Simvastatin users (ref.- rosuvastatin users)** | | | | | | |
|  | **Multiple imputations model** | | | **Incident users** | | |
|  | Coeff. | CI | p-value | Coeff. | CI | p-value |
| Average | -0.03 | -0.09; 0.03 | 0.342 | 0.01 | -0.12; 0.36 | 0.344 |
| Per year | 0.35 | 0.09; 0.61 | 0.008 | -1.63 | -3.18; -0.07 | 0.040 |
| After three years | 1.03 | 0.26; 1.80 | 0.009 | -4.77 | -9.46; -0.07 | 0.047 |
| **Lipophilic statins users (ref.- hydrophilic statins users)** | | | | | | |
|  | **Multiple imputations model** | | | **Incident users** | | |
|  | Coeff. | CI | p-value | Coeff. | CI | p-value |
| Average | -0.01 | -0.04; 0.04 | 0.980 | 0.12 | -0.13; 0.35 | 0.370 |
| Per year | 0.09 | -0.23; 0.42 | 0.576 | -1.32 | -2.46; -0.18 | 0.024 |
| After three years | 0.28 | -0.70; 1.26 | 0.575 | -3.84 | -7.28; -0.41 | 0.028 |
| **Fungal statins users (ref.- synthetic statins users)** | | | | | | |
|  | **Multiple imputations model** | | | **Incident users** | | |
|  | Coeff. | CI | p-value | Coeff. | CI | p-value |
| Average | -0.12 | -0.05; 0.01 | 0.152 | 0.01 | -0.07; 0.10 | 0.751 |
| Per year | 0.27 | -0.02; 0.56 | 0.066 | -0.61 | -1.19; -0.04 | 0.036 |
| After three years | 0.79 | -0.06; 1.65 | 0.070 | -1.83 | -3.55; -0.12 | 0.037 |
| **Statin users (ref.- non statin lipid-lowering medication users)** | | | | | | |
|  | **Multiple imputations model** | | | **Incident users** | | |
|  | Coeff. | CI | p-value | Coeff. | CI | p-value |
| Average | -0.01 | -0.08; 0.07 | 0.926 | -0.03 | -0.10; 0.05 | 0.516 |
| Per year | -0.63 | -2.05; 0.79 | 0.387 | -0.79 | -1.88; 0.31 | 0.158 |
| After three years | -1.89 | -6.11; 2.33 | 0.382 | -2.38 | -5.57; 0.89 | 0.154 |

Sensitivity analysis, multiple imputations of MMSE model and incident users. Linear mixed-effects regression model with inverse probability weighting, adjusted model for selected demographic characteristics, comorbidities and comedication (details in methods). Coefficient for the point-wise difference in MMSE points over time between comparison groups, 95% CI and two-sided p-values are reported. Incident users were defined as drug users who did not take out any drug prescription of statins during twelve months before six-month period preceding each SveDem entry date.

**Supplementary table** **7** Characteristics of incident users

|  | **All users**  **(n= 844)** | **Atorvastatin users**  **(n= 267)** | **p-value^1^** | **Simvastatin users**  **(n= 557)** | **p-value^2^** | **Rosuvastatin users**  **(n= 18)** |
| --- | --- | --- | --- | --- | --- | --- |
| MMSE score at baseline | 21.1 (4.6) | 20.5 (4.4) | 0.01 | 21.4 (4.7) | 0.79 | 21.1 (3.9) |
| Age at baseline | 76.7 (7.0) | 76.9 (7.0) | 0.63 | 76.6 (7.1) | 0.58 | 77.6 (4.7) |
| Women | 496 (58.8) | 154 (57.7) | 0.56 | 333 (59.8) | 0.72 | 10 (55.6) |
| Living arrangements  With another adult  Alone  Nursing home | 491 (58.2)  310 (36.7)  41 (4.9) | 151 (56.6)  107 (40.1)  9 (3.4) | 0.28 | 329 (59.1)  196 (35.2)  30 (5.4) | 0.87 | 9 (50.0)  8 (44.4)  1 (5.6) |
| Type of dementia diagnostic unit  Special memory clinic  Primary care | 600 (72.8)  224 (27.2) | 161 (60.3)  106 (39.6) | <0.001 | 439 (78.8)  118 (21.2) | 0.92 | 14 (77.8)  4 (22.2) |
| Ischemic stroke | 115 (13.6) | 40 (15.0) | 0.24 | 67 (12.0) | <0.001 | 7 (38.9) |
| Hemorrhagic stroke | 9 (1.1) | 1 (0.4) | 0.23 | 7 (1.3) | 0.13 | 1 (5.6) |
| Myocardial infarction | 71 (8.4) | 21 (7.9) | 0.85 | 46 (8.3) | 0.04 | 4 (22.2) |

Data are presented as mean (SD) for continuous measures and n (%) for categorical measures.

p-value^1^, atorvastatin vs simvastatin users; p-value^2^, rosuvastatin vs simvastatin users
